# Development and Prospects of Multi-Domain Geomagnetic Navigation and Positioning Technologies

**DOI:** 10.3390/s26185970

**Published:** 2026-09-21

**Authors:** Libo Zhu, Houpu Li, Bo Zhu, Bing Liu, Jineng Ouyang, Lei Xu, Shaofeng Bian, Fujiang Liu

**Affiliations:** 1 Naval University of Engineering, Wuhan 430033, China; 2 91937 Troops, Zhoushan 316000, China; 3 Naval Academy, Tianjin 301309, China; 4 China University of Geosciences, Wuhan 430033, China

**Keywords:** geomagnetic navigation, geomagnetic matching, geomagnetic filtering, bio-inspired navigation, multi-domain geomagnetic applications

## Abstract

To address the pressing demand for autonomous navigation in GNSS-denied environments, this paper systematically reviews the development status and future trends of multi-domain geomagnetic navigation and positioning technologies. First, from the perspectives of scalar and vector measurement systems, the technical characteristics of main-stream magnetometers, including optically pumped magnetometers, proton precession magnetometers, and fluxgate magnetometers, are comparatively analyzed, and the key parameters of several high-resolution global and regional geomagnetic field models are summarized. Then, geomagnetic navigation algorithms are classified into four categories, namely geomagnetic matching, geomagnetic filtering, bio-inspired navigation, and AI-assisted methods, and the core principles and technical features of each category are discussed in detail. On this basis, representative application scenarios in aviation, marine and underwater systems, and indoor environments are examined, with emphasis on their technical bottlenecks, representative experiments, and development trends. Finally, future directions are outlined from three aspects: high-precision magnetic data acquisition and high-resolution reference map construction, deep fusion of multi-source information and intelligent algorithm innovation, and engineering deployment and industrialization. This paper aims to provide theoretical references and technical support for the development of passive autonomous multi-domain navigation capabilities.

## 1. Introduction

The current navigation architecture remains heavily dependent on Global Navigation Satellite Systems (GNSS), rendering it particularly vulnerable in increasingly complex electromagnetic confrontation environments. Repeated episodes of “navigation warfare” in conflicts ranging from the Iraq War and the Kosovo conflict to the Russia–Ukraine conflict and the U.S.–Israel–Iran confrontation have fully exposed the susceptibility of GNSS signals to jamming, spoofing, and direct attacks. Consequently, in GNSS-denied environments such as deep-sea, underground, indoor, and heavily jammed airspace, ensuring precise navigation capability for mobile platforms including unmanned aerial vehicles, aircraft, and underwater vehicles, and preventing mission failure caused by navigation degradation, has become a major scientific and engineering challenge that requires urgent attention [1,2,3,4,5,6].

In this context, autonomous navigation technologies such as terrain-aided navigation [7], gravity-aided navigation [8], visual navigation [9], and geomagnetic navigation [10] have advanced rapidly. However, each approach remains constrained by specific application conditions. Terrain matching and visual navigation are prone to failure in feature-sparse regions such as plains, oceans, and deserts, and are further limited by adverse weather conditions, particularly low visibility. Although gravity navigation offers long-term stability, its performance depends on high-precision gravimetric sensing equipment, which substantially increases system cost. Visual navigation, in particular, exhibits a marked decline in localization reliability in low-light or texture-poor scenes [11,12].

By contrast, the geomagnetic field is a natural physical field of the Earth. Geomagnetic navigation exploits the one-to-one correspondence between geomagnetic features and geographic location to enable positioning, offering distinct advantages such as passivity, no radiation, strong stealth, and round-the-clock, all-weather, global coverage [13,14,15]. From a technological perspective, geomagnetic navigation was first experimentally validated at Lockman Air Force Base in the United States, and the emergence of the geomagnetic anomaly matching system (MAGCOM) in the 1960s marked a major breakthrough in geomagnetic matching techniques [16,17]. In recent years, the MAGNAV project jointly carried out by the U.S. Air Force and MIT [18], the AQNav system developed by SandboxAQ [19,20], and the geomagnetic navigation system developed by the Australian company Q-CTRL [21] have achieved successive breakthroughs, fully demonstrating the performance potential of this technology in navigation applications.

Over the past several decades, geomagnetic navigation has followed a clear technological trajectory. From early geomagnetic contour matching, which relied on batch-processing algorithms for position estimation, to geomagnetic filtering algorithms that incorporated geomagnetic information into the navigation mechanism for continuous trajectory correction, and further to the recent introduction of neural networks, the field has progressed rapidly from manually designed feature extraction and matching rules toward data-driven autonomous cognition and decision-making [22,23,24]. Along with these technological advances, geomagnetic navigation has also expanded its application scope in multiple dimensions, comprehensively covering aviation, marine and underwater systems, and indoor environments.

This paper first reviews the current status of geomagnetic field measurement and modeling, then summarizes several mainstream geomagnetic navigation algorithms, and subsequently discusses their applications in multi-domain scenarios in detail. Finally, it concludes by analyzing the development trends and future prospects of geomagnetic navigation.

## 2. Current Status of Geomagnetic Field Measurement and Modeling

The implementation of geomagnetic navigation relies on two fundamental prerequisites: high-precision, highly stable real-time geomagnetic field measurement, and geomagnetic reference maps with high spatial resolution and strong timeliness. The former determines the quality of the observational data used by the navigation system, whereas the latter directly constrains the theoretical upper bound of positioning accuracy. As application platforms have expanded from ships and manned aircraft to unmanned aerial vehicles, underwater vehicles, and even smartphones, sensor technologies have evolved along two parallel tracks, namely scalar and vector measurement systems. Meanwhile, geomagnetic field modeling has also advanced within the global reference-field framework toward local refinement and multi-source data fusion.

### 2.1. Geomagnetic Field Measurement

Geomagnetic sensing instruments form the physical basis by which geomagnetic navigation acquires environmental prior features and real-time observations. Their performance not only determines the quality of the reference geomagnetic maps, but also directly constrains the accuracy of real-time matching-based positioning. As carrier platforms have evolved, sensor hardware has also undergone substantial changes. On the one hand, measurement modalities have gradually expanded from a single scalar quantity to multidimensional measurements, including vector, gradient, and tensor fields. On the other hand, manufacturing processes have shown a clear trend from large-scale precision instruments toward miniaturized, low-power chips [13,25].

Current mainstream magnetometers can be divided into two categories: scalar magnetometers and vector magnetometers. Scalar magnetometers are represented by optically pumped magnetometers and proton precession magnetometers. Optically pumped magnetometers are based on Zeeman splitting of helium and alkali-metal atoms, such as potassium, rubidium, and cesium, in an external magnetic field, and are developed by combining optical pumping and magnetic resonance techniques. They are characterized by high accuracy, good stability, and reliable data quality [26,27,28]. Among them, cesium optically pumped magnetometers are the most mature. Representative commercial products include the CS-3 (Scintrex Ltd., Concord, ON, Canada) [29,30], the G-882 (Geometrics, Inc., San Jose, CA, USA) [27], the MG02 and MG03 (Nanjing Fangzhiyu Technology Co., Ltd., Nanjing, China) [12], and the LCT-2A (Hangzhou MicroQuantum Technology Co., Ltd., Hangzhou, China). Rubidium optically pumped magnetometers are compact, low-power, and cost-effective, and can serve as alternatives to cesium systems under budget constraints. The VRuM miniaturized rubidium optically pumped magnetometer designed by the University of Colorado Boulder, Boulder, CO, USA, can achieve synchronized vector measurement capability on top of conventional scalar measurement [31], and the miniaturized rubidium optically pumped magnetometer developed by Jiataike (Shenzhen) Technology Co., Ltd., Shenzhen, China, can reach a sensitivity of 5 pT/√Hz. Helium optically pumped magnetometers include the domestic GB-4A magnetometer and GB-10 magnetometer (The 715th Research Institute of China State Shipbuilding Corporation Limited, Hangzhou, China) [29,30,32], although helium-based systems are generally larger and more power-consuming. High-precision potassium optically pumped magnetometers offer the highest accuracy, such as the GSMP-35 (GEM Systems Inc., Markham, ON, Canada) [33,34,35], with a resolution of 0.1 pT, but they are often more expensive.

Proton magnetometers implement total-field absolute measurement of the geomagnetic field based on the Larmor free-precession principle. Their advantages include direct acquisition of the absolute scalar value of the geomagnetic field without directional alignment, low equipment cost, simple maintenance, and long-term data stability and reliability. Among them, the CZM series developed by Beijing Geophysical Instrument Factory, Beijing, China, is widely used in China’s geomagnetic observatories and field magnetic surveys [36]. The JOM-5SF Overhauser magnetometer developed by Jizhi Magnetism Measurement Technology (Changchun) Co., Ltd., Changchun, China, achieves an absolute accuracy of 0.1 nT and a resolution of 0.001 nT [37]. The GSM-19 Overhauser-type proton magnetometer from GEM Systems Inc., Markham, ON, Canada, is a commonly used instrument for international diurnal observatories, with an absolute accuracy of 0.1 nT and a resolution of 0.01 nT [12]. The G-857 proton magnetometer from Scintrex Ltd., Concord, ON, Canada [38], offers a large gradient tolerance and is suitable for both portable magnetic surveys and base-station observations. However, proton magnetometers are generally relatively large and have low sampling rates [32]. They are therefore typically used in applications that require high absolute accuracy but place comparatively looser demands on timeliness, such as long-term geomagnetic observatory time-series monitoring, ground diurnal stations for aeromagnetic and marine magnetic surveys, and earthquake precursor observation.

Fluxgate magnetometers are the representative vector magnetometers in geomagnetic observation because of their high sensitivity, low power consumption, and good stability. Mature products include the Mag-13 (Bartington Instruments Ltd., Witney, UK) [33], the MFG-2S (MAGSON GmbH, Berlin, Germany), and the Mag-03 series fluxgate sensors (Xi’an Huashun Measurement Equipment Co., Ltd., Xi’an, China) [39,40].

Magnetoresistive magnetometers realize three-dimensional geomagnetic vector measurement based on the magnetoresistive effect. Featuring compact size, low power consumption and favorable MEMS integrability, they serve as mainstream vector magnetometers for geomagnetic-aided navigation of miniature carriers, covering three technical branches of AMR, GMR and TMR [13,41]. Typical commercial products include the Honeywell HMC1021S AMR magnetometer (Honeywell International Inc., Charlotte, NC, USA), the NVE AA002-02E GMR magnetometer (NVE Corporation, Eden Prairie, MN, USA) [42], and Bosch BMM350 TMR magnetometer (Bosch Sensortec GmbH, Reutlingen, Germany) [43]. These sensors are widely applied in inertial/geomagnetic integrated navigation for ground robots, smartphones and pedestrian dead reckoning systems, mainly providing heading information.

In summary, magnetometer selection requires a multi-dimensional trade-off analysis based on the specific mission. The primary considerations include the type of geomagnetic parameter to be measured (scalar, vector, or gradient), the sampling and temporal resolution requirements determined by the carrier’s motion state, the environmental adaptability under complex operating conditions such as temperature variation and vibration, the hardware constraints of the payload platform regarding size, power consumption, and weight, as well as equipment acquisition cost and post-deployment maintenance. Optically pumped magnetometers, owing to their high temporal resolution, are more suitable for geomagnetic measurement scenarios involving high-speed carriers such as aircraft, but certain models require stringent temperature compensation and high-end products are expensive. Proton magnetometers are limited by their sampling rates and are poorly adapted to apply to high-speed dynamic measurements, but their stable absolute measurement capability and cost advantages still make them important for geomagnetic reference observation and diurnal observatories. The core advantage of fluxgate magnetometers is their ability to directly output three-component vector magnetic field data, but their sensitivity, resolution, and sampling rates are relatively limited overall, making them suitable for measurement tasks that require vector field features and also for carrier magnetic interference compensation. Benefiting from superior miniaturization, low power consumption and high integration, magnetoresistive magnetometers are well suited for auxiliary navigation applications of lightweight and low-cost carriers, such as smartphones, small unmanned aerial vehicles, ground robots and pedestrian dead reckoning platforms. However, constrained by inherent magnetic hysteresis, temperature drift and limited measurement precision, these sensors suffer from relatively weak magnetic interference resistance, which limits their applicability to high-precision and high-stability geomagnetic matching positioning and refined geomagnetic observation tasks. Table 1 compares the key performance indicators of commonly used magnetometers.

### 2.2. Current Status of Geomagnetic Field Modeling

Geomagnetic reference maps serve as the physical information carrier of geomagnetic navigation, and their spatial resolution and field characterization accuracy directly determine system positioning performance. At present, publicly available geomagnetic field models mainly include main-field models and magnetic anomaly field models. Common main-field models include the International Geomagnetic Reference Field (IGRF-14) [44], the World Magnetic Model (WMM-2025) [45], and CHAOS-8 [46]. However, global main-field models have relatively low spatial resolution and are unable to resolve regional short-wavelength magnetic anomalies. As a result, they cannot be used alone as geomagnetic navigation reference maps, although they can be applied in the process of deriving magnetic anomalies from measured total-field data, i.e., by removing the long-wavelength components. For example, Thébault et al. used UAV aeromagnetic data combined with the CHAOS model and employed rectangular harmonic analysis (RHA) to rapidly construct scalar and vector magnetic anomaly maps [47]. In addition, models such as WDMAM [48], EMAG2v3 [49], and NGDC-720 [50] provide finely gridded regional datasets. Magnetic data publicly released by the National Oceanic and Atmospheric Administration National Geophysical Data Center (NOAA NGDC, https://www.ngdc.noaa.gov/, accessed on 15 May 2026), INTERMAGNET (https://www.intermagnet.org/, accessed on 15 May 2026), and the China Meridian Project network (https://data.meridianproject.ac.cn/, accessed on 15 May 2026) also constitute important geomagnetic map resources for regional high-resolution geomagnetic navigation. Hui Zheng et al. (2013) [51] used gravity anomaly grid data (GAG2) and EMAG2 grid data as a background database, and adopted a multi-model adaptive estimation method for gravity- and magnetic-aided navigation, thereby effectively improving the accuracy and reliability of the aided navigation system. Zhong et al. (2020) [52] used EMAG2 grid data as the reference map, selected the South China Sea as the simulation test area, and achieved high matching accuracy. Q-CTRL in Australia, relying entirely on publicly available magnetic anomaly maps released by Geoscience Australia (https://ecat.ga.gov.au/geonetwork/, accessed on 15 May 2026), has demonstrated geomagnetic navigation performance far beyond that of strategic-grade inertial navigation systems by deploying quantum sensors on airborne and ground platforms [21]. Table 2 lists several high-resolution global and regional anomaly-field models.

In terms of modeling methods, mainstream regional modeling approaches can be broadly divided into numerical fitting methods and mathematical-physical modeling methods. Numerical fitting methods use a set of functions or polynomials to model the fine-scale structure of the geomagnetic field in local regions. Common functions include Taylor polynomials, Legendre polynomials, Chebyshev polynomials, and spline functions. Such methods are convenient to use and can represent the spatial distribution of the geomagnetic field reasonably well. However, when the truncation order is high, the computational burden becomes substantial, and the methods do not strictly satisfy the theoretical constraints of geomagnetic potential theory [53,54,55]. Mathematical-physical modeling methods are based on mathematical physics theory and include rectangular harmonic analysis [56], spherical cap harmonic analysis [57], modified spherical cap harmonic analysis [58], and the equivalent source method [59]. These methods satisfy the physical constraints imposed by geomagnetic potential theory and can construct both scalar and vector geomagnetic fields while effectively representing the three-dimensional structure of the geomagnetic field. However, their computation is relatively complex. Therefore, the choice of modeling method should be made by considering factors such as data volume, region size, and required modeling resolution.

**Table 2:** Selected high-resolution global and regional Geomagnetic field models.

Model/ Geomagnetic Map Name	Model/ Geomagnetic Map Type	Degree/Order	The Max Spatial Resolution/km	References	Reference Sites
EMAG2v3	Global model	Grid 2 arcminute	3.7	[49]	https://www.ncei.noaa.gov/products/earth-magnetic-model-nomaly-grid-2, accessed on 23 May 2026
NGDC-720	Global model	SH 720	56	[50]	https://geomag.colorado.edu/index.php/ngdc-720-lithospheric-magnetic-model, accessed on 23 May 2026
NGDC-720V4QT	Global model	SH 740	54	[60]	https://libyc.nudt.edu.cn/https/443/org/zenodo/yitlink/records/15641536, accessed on 23 May 2026
EMM2017	Global model	SH 790	51	\	https://www.ncei.noaa.gov/products/enhanced-magnetic-model, accessed on 23 May 2026
HDGM2026	Global model	SH 1040	38.5	\	https://www.ncei.noaa.gov/products/high-definition-geomagnetic-model, accessed on 23 May 2026
WDMAM (EMAG3)	Global model	Grid 3 arcminute	5.6	[48]	https://geomag.org/models/wdmam.html, accessed on 23 May 2026
CUG_GLMFM3Dv1	Regional model	R-SCHA 1500	6	[61]	\
SH-1050	Regional model	SH 1050	40	[62]	https://zenodo.org/record/5546528, accessed on 23 May 2026
Total Magnetic Intensity (TMI) Grid of Australia 2019—seventh edition	Regional geomagnetic map	Grid 0.05 arcminute	0.08	\	https://ecat.ga.gov.au/geonetwork/srv/api/records/f2e58161-24cf-42d2-b328-a9039f72113b, accessed on 23 May 2026

Note: The spatial resolution of a geomagnetic field model can be calculated as $L\approx 2\pi a/N_{\max}$, where a is the Earth’s reference radius and $N_{\max}$ is the model truncation degree [54]. One arc-minute can be approximated as one nautical mile. SH denotes spherical harmonic model, R-SCHA denotes modified spherical cap harmonic model, and Grid denotes a gridded model.

## 3. Geomagnetic Positioning and Navigation Algorithm Framework

The essence of geomagnetic navigation algorithms is to exploit the mapping relationship between the spatial distribution characteristics of the geomagnetic field and geographic location. By comparing real-time measurements with prior reference information, these algorithms estimate the current position of the platform or correct the accumulated error of an inertial navigation system. Depending on the information-processing strategy and the degree of dependence on prior geomagnetic maps, existing methods can be broadly classified into four categories: geomagnetic matching, geomagnetic filtering, bio-inspired navigation, and AI-assisted navigation. From the perspective of technological development, geomagnetic matching serves as the fundamental core of traditional geomagnetic navigation, which realizes position solving relying on geomagnetic map matching. As an upgraded technology derived from the integration requirement of geomagnetic matching and inertial navigation, geomagnetic filtering achieves continuous recursive correction of navigation states and remedies the defects of discrete positioning and error accumulation in conventional matching methods. Bio-inspired geomagnetic navigation breaks through the inherent limitation of reliance on prior geomagnetic databases, realizing the innovative expansion of benchmark-map-free autonomous navigation. Artificial intelligence-assisted geomagnetic navigation acts as a technical empowerment approach for complex dynamic scenarios, which can be embedded into the above three traditional algorithm frameworks to optimize overall navigation performance, representing a technological innovation and upgrading of conventional geomagnetic navigation theories. The four types of algorithms possess respective technical advantages and applicable scopes. In practical engineering applications, technical integration and method multiplexing among these algorithms are prevalent, which collectively form a relatively systematic algorithm framework for geomagnetic navigation. This section systematically elaborates on the core principles, technical characteristics, and development trajectory of these four categories.

### 3.1. Geomagnetic Matching Algorithms

**Figure 1:**
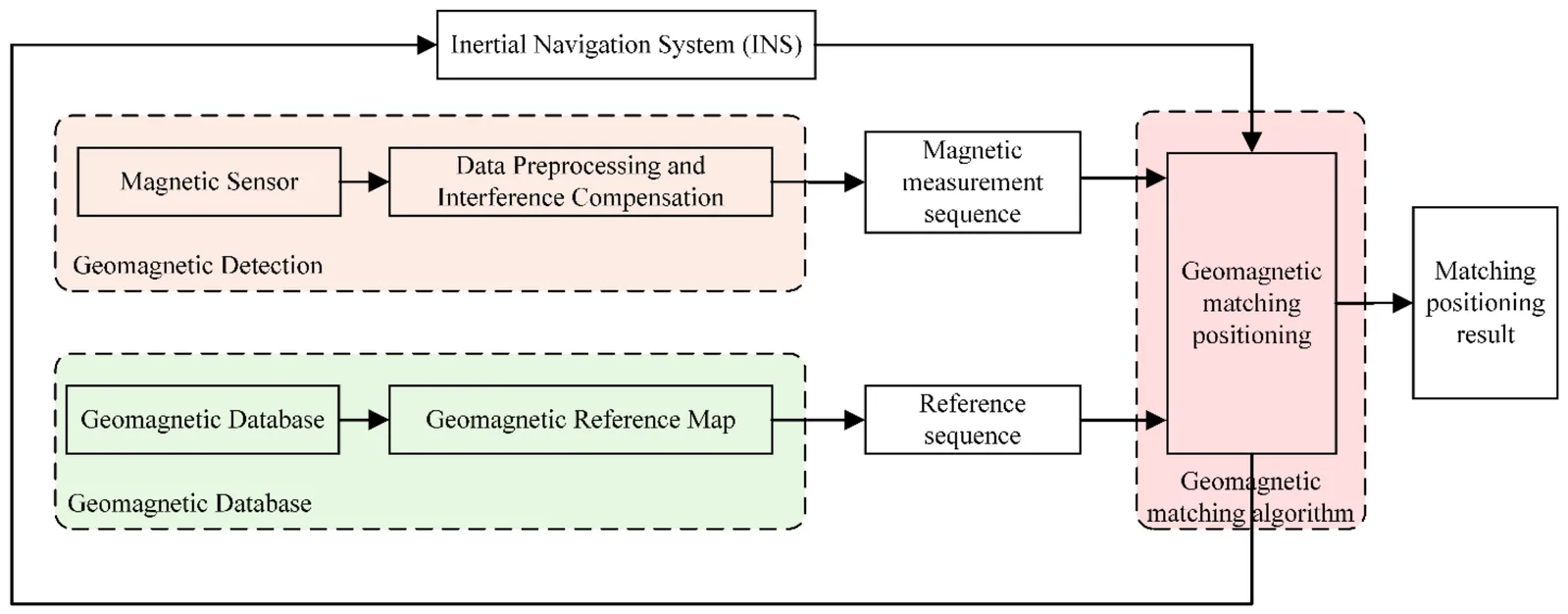
The basic principle diagram of geomagnetic matching navigation.

Geomagnetic matching is a method that, on the premise that the initial state of the platform is known, performs correlation analysis between a magnetic measurement sequence accumulated over a certain period by the sensors and a pre-established geomagnetic reference map, thereby obtaining the current position of the platform or a position correction term [4,15,63]. Figure 1 illustrates the basic principle of geomagnetic matching navigation.

As shown in Figure 1, geomagnetic matching navigation consists of three parts: geomagnetic sensing, the geomagnetic database, and the geomagnetic matching algorithm. Among the matching algorithms, the most representative ones are geomagnetic correlation matching (MAGCOM) and iterative closest contour point (ICCP). MAGCOM evolved from the terrain contour matching (TERCOM) algorithm. Its mechanism is to perform a grid-based translational search within the confidence region of the INS-indicated trajectory, thereby constructing multiple candidate trajectories. The magnetic field prediction sequence corresponding to each candidate trajectory is then correlated with the measured sequence from the magnetic sensor, and the most correlated candidate trajectory is selected as the matching result. MAGCOM has the advantages of a simple principle, broad applicability, and relatively large tolerance to initial errors. However, its matching process implicitly assumes that the platform moves in uniform straight-line motion at constant altitude, which to some extent limits the maneuvering freedom within the matching region. In addition, the algorithm applies only rigid translation to the INS-indicated trajectory to construct the matching template, which in essence assumes that the INS heading is unbiased and that only position bias exists. Therefore, it cannot effectively correct the velocity error or heading drift of the inertial navigation system. The ICCP algorithm originates from the iterative closest point (ICP) algorithm in the image matching field. Its core mechanism is to establish the optimal spatial transformation between the measured trajectory and the nearest geomagnetic contour through iterative optimization. Therefore, it can simultaneously correct the initial position error and heading deviation of the INS. ICCP offers relatively high accuracy; however, its strict convergence depends on three ideal assumptions: the magnetic sensor observations are error-free; the true position of the platform lies exactly on, or extremely close to, the contour corresponding to the measured magnetic value; and this true position is within the vicinity of the position indicated by the reference navigation system. To overcome the limitations of geomagnetic matching algorithms, a variety of improved methods have been proposed. In general, these methods are combinations of four elements: feature space, similarity metric, search space, and search strategy [63,64,65]. Among them, similarity metrics can quantitatively describe the correlation among the actual measured trajectory, the reference-map trajectory, and the matched position. Commonly used correlation criteria for evaluating such correlations are documented in References [15,66].

### 3.2. Geomagnetic Filtering Algorithms

Geomagnetic filtering is a recursive state-estimation approach that uses the inertial navigation system (INS) as the time reference frame and the geomagnetic field as an external source of observations. Its basic principle is to construct a measurement residual from the difference between the magnetic field predicted at the INS-estimated position on the geomagnetic reference map and the magnetic field measured by the magnetometer. A joint filtering model is then established by combining the geomagnetic observation equation with the navigation-state equation. Through recursive state prediction and measurement update, the method enables real-time correction of states such as position, velocity, attitude, and sensor errors, thereby embedding geomagnetic spatial constraints into the INS error-propagation process and suppressing the divergence of navigation errors over time.

**Figure 2:**
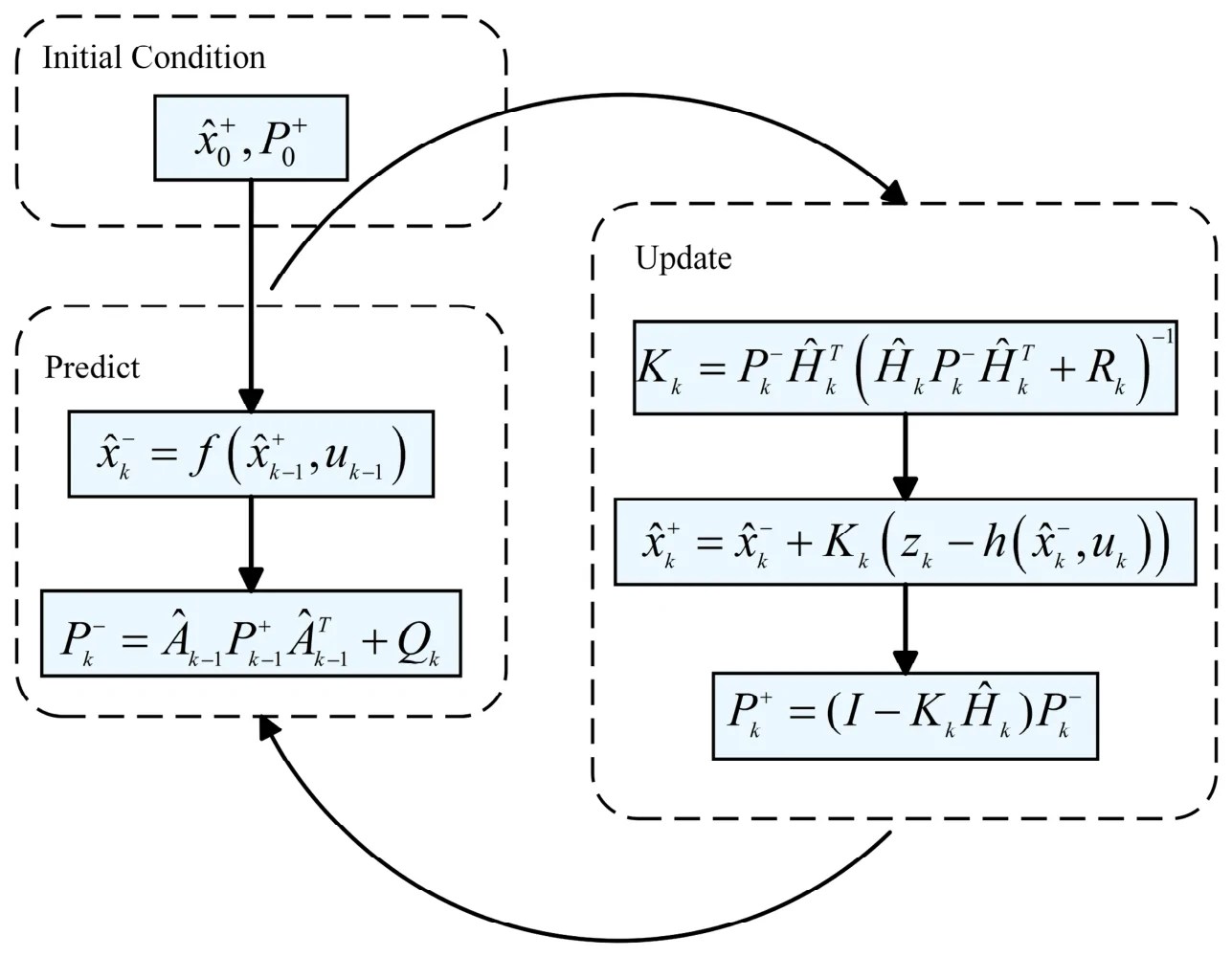
The general recursive estimation framework of Kalman filtering.

The main filtering algorithms used in geomagnetic filtering navigation include the Kalman filter (KF) [67,68], extended Kalman filter (EKF) [69,70], unscented Kalman filter (UKF) [71,72], particle filter (PF) [73,74], adaptive Kalman filter (AKF) [75,76], and federated filter (FF) [77,78]. Figure 2 illustrates the generic recursive estimation framework of the Kalman filter, which is centered on a priori prediction and a posteriori measurement update. In the prediction step, the state estimate is propagated according to the system state-transition model, and the error covariance is propagated accordingly. In the update step, the Kalman gain feeds the geomagnetic measurement residual back into the state correction, thereby recursively refining the error covariance. The EKF extends the standard KF to nonlinear systems by using a Taylor-series expansion to locally linearize the nonlinear geomagnetic observation equation and the INS state equation. However, this generic recursive framework does not fully capture the core mechanisms of the other mainstream algorithms. The UKF replaces linearization with the unscented transform and uses a deterministic sigma-point set to accurately propagate the statistics through nonlinear dynamics, thereby improving estimation accuracy and stability over the EKF. The PF approximates the posterior state distribution with a set of random particles via sequential importance sampling, thereby relaxing the Gaussian assumption and making it suitable for strongly nonlinear and non-Gaussian environments. The AKF adaptively estimates and updates the characteristics of system noise and measurement noise online, thereby improving robustness under model mismatch and geomagnetic disturbances. The FF adopts a hierarchical architecture in which multiple local filters process heterogeneous information in parallel and are then fused and reset by a master filter, enabling multi-level fault-tolerant fusion of geomagnetic, inertial, and other navigation sources. Table 3 compares the common geomagnetic filtering algorithms [67,79].

In Figure 2, ${\hat{x}}_{0}^{+}$ and $P_{0}^{+}$ denote the initial state vector and initial error covariance matrix, respectively. The state vector ${\hat{x}}_{0}^{+}$ may include position, velocity, attitude, accelerometer bias, barometer bias, and other state variables. In the prediction stage, the two equations correspond to the state propagation model and the covariance prediction model, where ${\hat{x}}_{k}^{-}$ and ${\hat{x}}_{k-1}^{-}$ denote the navigation state at the current and previous instants, respectively; f denotes the state-transition function; $P_{k}^{-}$ and $P_{k-1}^{+}$ denote the current and previous error covariance matrices, respectively; ${\hat{A}}_{k-1}$ is the state-transition matrix; and $Q_{k}$ is the process-noise covariance matrix. In the update stage, the state and covariance are corrected accordingly. Here, $K_{k}$ is the Kalman gain matrix used to balance the prior estimate and the measurement information; $R_{k}$ is the measurement-noise covariance matrix; ${\hat{H}}_{k}^{T}$ is the observation matrix; $z_{k}$ is the measurement; h(⋅) describes the measurement model; and I is the identity matrix of compatible dimension.

### 3.3. Bio-Inspired Navigation Algorithms

**Figure 3:**
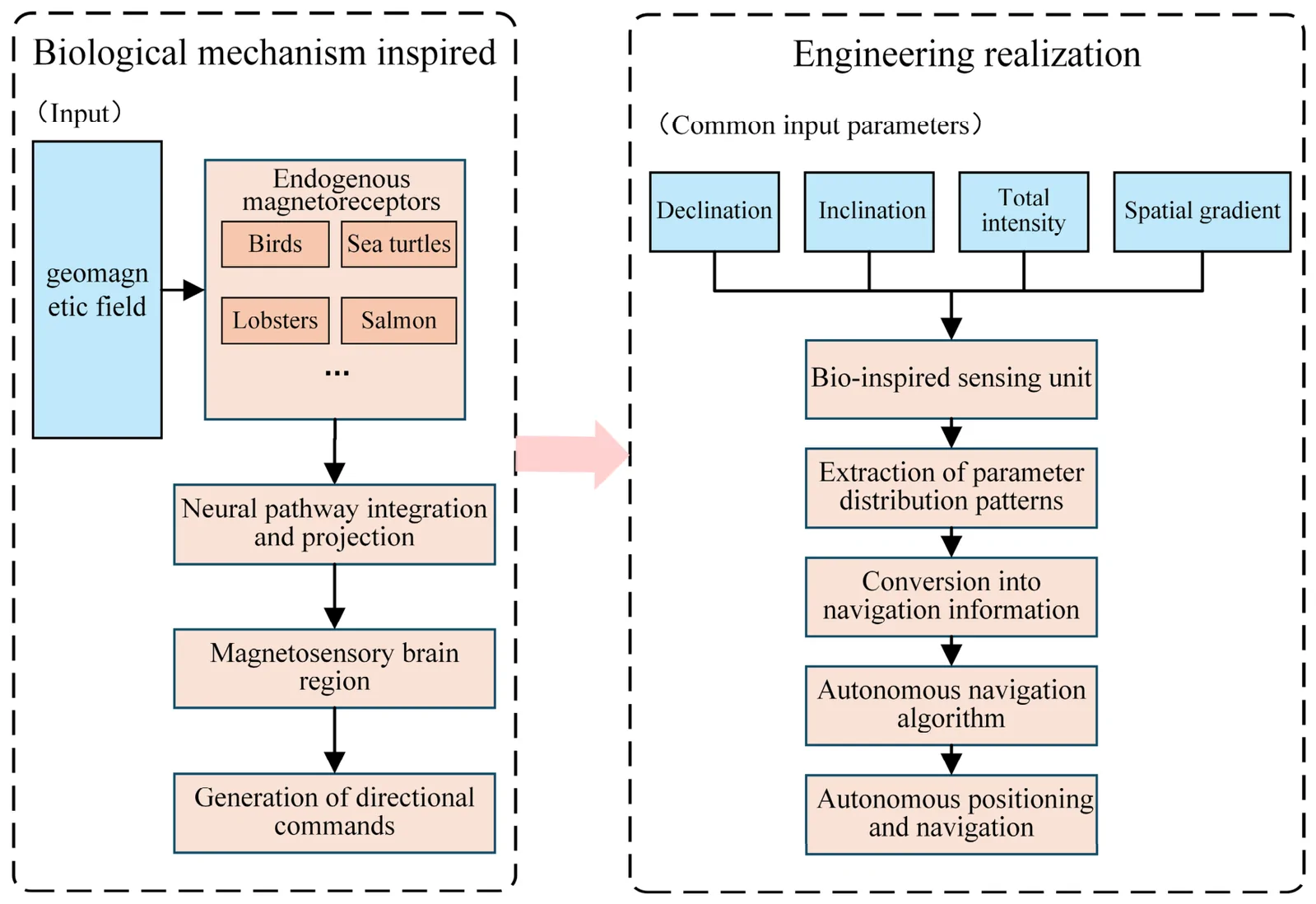
Basic principle of bio-inspired navigation.

Bio-inspired navigation is a navigation paradigm that emulates biological geomagnetic perception mechanisms and enables autonomous long-range navigation without requiring a prior digital geomagnetic database in the engineering sense. It mainly draws on the physiological mechanisms by which lobsters [10], sea turtles [80], salmon [81], and birds [82,83] perceive geomagnetic information through endogenous magnetoreceptors, and then integrate this information via neural pathways and project it to magnetosensory brain regions to generate directional commands. By converting the spatial distribution patterns of multiple geomagnetic parameters, such as magnetic declination, magnetic inclination, total intensity, and their spatial gradients, into navigational information, the carrier can accomplish positioning and path planning without relying on a pre-stored magnetic anomaly reference map [84,85]. Figure 3 illustrates the basic principle of bio-inspired navigation.

From a bio-inspired perspective, the geomagnetic field is a complex spatial information field that contains multiple characteristic parameters, such as total intensity, magnetic declination, and magnetic inclination. These parameters not only exhibit significant regional variability in space, but each also follows a specific spatial distribution law. As shown in Figure 3, autonomous navigation based on geomagnetic parameters essentially relies on continuous platform motion to drive the geomagnetic parameters at the current position to gradually converge to the prescribed values at the target position. Therefore, from the perspective of multi-objective optimization, the system uses bio-inspired navigation algorithms to estimate errors in real time and output motion commands, thereby guiding the platform to perform continuous heuristic search and state update in an unknown geomagnetic environment and ultimately achieving efficient autonomous navigation toward the target point.

From the application-oriented perspective, bionic geomagnetic navigation is developed to address the practical bottleneck that conventional geomagnetic matching navigation heavily relies on full-coverage prior geomagnetic reference maps [84,86,87]. Conventional geomagnetic matching navigation requires pre-surveyed geomagnetic measurements over large-scale regions, which is often difficult to achieve in environments such as oceans and remote uninhabited areas. In contrast, bionic geomagnetic navigation only requires multi-parameter geomagnetic features at the target position, without pre-storing a global geomagnetic database. Relying on real-time geomagnetic observations collected during carrier movement, it gradually approaches the target position through heuristic search iterations. Nevertheless, this technology comes with inherent application constraints. In geomagnetically quiet zones with weak geomagnetic gradients, the heuristic search is highly prone to local optima. Its navigation performance is sensitive to geomagnetic environmental characteristics. Moreover, limited by the iterative optimization mechanism, its navigation performance degrades significantly in high-dynamic mission scenarios that demand stringent instantaneous positioning outputs. Accordingly, bionic geomagnetic navigation is more suitable for medium-to-long-range autonomous missions where iterative-search procedures are permissible. It is commonly fused with other navigation means such as inertial navigation to further improve the overall system robustness under complex environments.

### 3.4. Artificial Intelligence-Assisted Algorithms

In recent years, advances in computer technology—particularly big data and high-performance computing—have enabled the widespread application of artificial intelligence (AI) algorithms across diverse fields, and are now driving geomagnetic navigation toward an intelligent navigation paradigm. Depending on the depth of AI involvement in the geomagnetic information-processing chain, existing research can be categorized into four aspects: signal enhancement, feature reconstruction, decision optimization, and system-architecture integration.

At the signal level, neural network methods have been introduced into geomagnetic data prediction, as well as into the magnetic compensation and signal separation of magnetometer measurements. In geomagnetic data prediction, because measurement data are readily corrupted by environmental and observational-system disturbances, prediction techniques can effectively safeguard data quality and optimize geomagnetic information management. Yao (2018) [88] employed a BP neural network to reconstruct geomagnetic observations, effectively mitigating the adverse effects of data-quality problems on prediction accuracy. Liu et al. (2019) [89] further proposed a geomagnetic data prediction method that combines machine learning and deep learning techniques, demonstrating high adaptability and prediction accuracy across different datasets. Lu (2021) [90] projected temporal and spatial information onto circular coordinates and applied a BP neural network, further improving the prediction accuracy of geomagnetic variation fields. In the magnetic compensation and signal separation of magnetometer data, the Tolles–Lawson (T–L) aeromagnetic compensation model remains the most classical framework; however, the coefficient matrix of the model frequently suffers from severe multicollinearity. Numerous researchers have therefore introduced neural networks to improve upon the conventional compensation model. Williams et al. (1993) [91] were among the first to apply neural network methods to solve aeromagnetic compensation coefficients, successfully separating the airframe-induced interference field. Gnadt et al. (2022) [92] designed several neural network approaches—including conventional data-driven, adaptive filtering, and T–L physics-embedded variants—and effectively extracted clean magnetic signals in manned-aircraft aeromagnetic experiments. In the field of indoor geomagnetic navigation, Hu et al. (2020) [93] applied a one-dimensional convolutional neural network (1D CNN) to the screening and classification of indoor geomagnetic data, successfully distinguishing magnetically disturbed measurements from genuine geomagnetic field data and significantly improving the accuracy of heading estimation.

At the feature and matching level, deep learning is reshaping the core logic of geomagnetic matching. Conventional matching methods based on correlation criteria or contour constraints tend to converge to local optima or suffer from multi-solution ambiguity in weakly featured regions, whereas neural networks can recast geomagnetic navigation as a pattern recognition or regression estimation problem through nonlinear mapping. Zhou et al. (2010) [94] introduced the probabilistic neural network (PNN) into geomagnetic matching, formulating position determination within a statistical pattern recognition framework. Kim et al. (2019) [34] combined a convolutional neural network with normalized cross-correlation to achieve rapid screening of candidate regions. Chen et al. (2023) [95] further proposed a two-stage neural network-based geomagnetic vector navigation method that classifies geomagnetic vectors and their feature information, achieving high matching success even in weakly gradient regions. The geomagnetic gradient multi-attention neural network (GGMA) proposed by Yang et al. (2024) [96] extracts spatiotemporal features of geomagnetic gradients and effectively suppresses navigation failure under magnetic anomaly interference.

At the decision and search level, reinforcement learning and evolutionary computation provide new methodologies for navigation path planning and search-space optimization. To address bottlenecks such as the premature convergence of conventional particle swarm optimization (PSO) and the sensitivity of geomagnetic matching to initial errors, Ji et al. (2017) [97] proposed a simulated-annealing-improved particle swarm optimization geomagnetic matching algorithm (SAPSO), which enhances matching accuracy and noise immunity through constrained initialization and adaptive parameter adjustment. Wang et al. (2020) [98] developed a geomagnetic navigation algorithm for autonomous underwater vehicles (AUVs) based on a deep double-Q-network with an adaptive search space, achieving efficient and stable round-trip navigation in simulation without a priori geomagnetic maps. Li et al. (2024) [99] further integrated deep reinforcement learning with simulated annealing, enabling effective path planning and geomagnetic map construction and thereby significantly improving the navigation performance of autonomous underwater vehicles in unknown environments.

At the system-architecture level, hybrid intelligent frameworks that incorporate AI are emerging as a frontier trend in geomagnetic navigation. Cuenca et al. (2025) [74] designed an intelligent geomagnetic navigation framework integrating a generative adversarial network (GAN), a deep Q-network (DQN), and a Rao–Blackwellized particle (RBP) filter. Within this framework, the GAN addresses map construction in data-sparse regions, while the DQN adaptively adjusts the observation covariance matrix parameters of the RBP filter, forming a complete geomagnetic architecture that effectively enhances filtering robustness and navigation accuracy in dynamic environments.

In summary, AI-assisted geomagnetic navigation is evolving from the optimization of isolated processing stages toward the construction of complete geomagnetic navigation system frameworks. Empowered by deep learning and reinforcement learning, these techniques effectively alleviate long-standing challenges such as signal interference, weak-feature matching, and dynamic decision-making in complex environments, significantly enhancing both the accuracy and the environmental robustness of navigation systems.

## 4. Geomagnetic Navigation Applications in Multi-Domain Scenarios

In recent years, with the rapid development of quantum magnetometers, miniaturized magnetic sensors, and intelligent algorithms, geomagnetic navigation has achieved significant progress from theoretical validation to engineering trials across three major fields: aviation, marine and underwater systems, and indoor environments. This section reviews the technical schemes, representative experiments, and development trends of geomagnetic navigation systems in these three typical application scenarios.

### 4.1. Aviation Platform Applications

**Figure 4:**
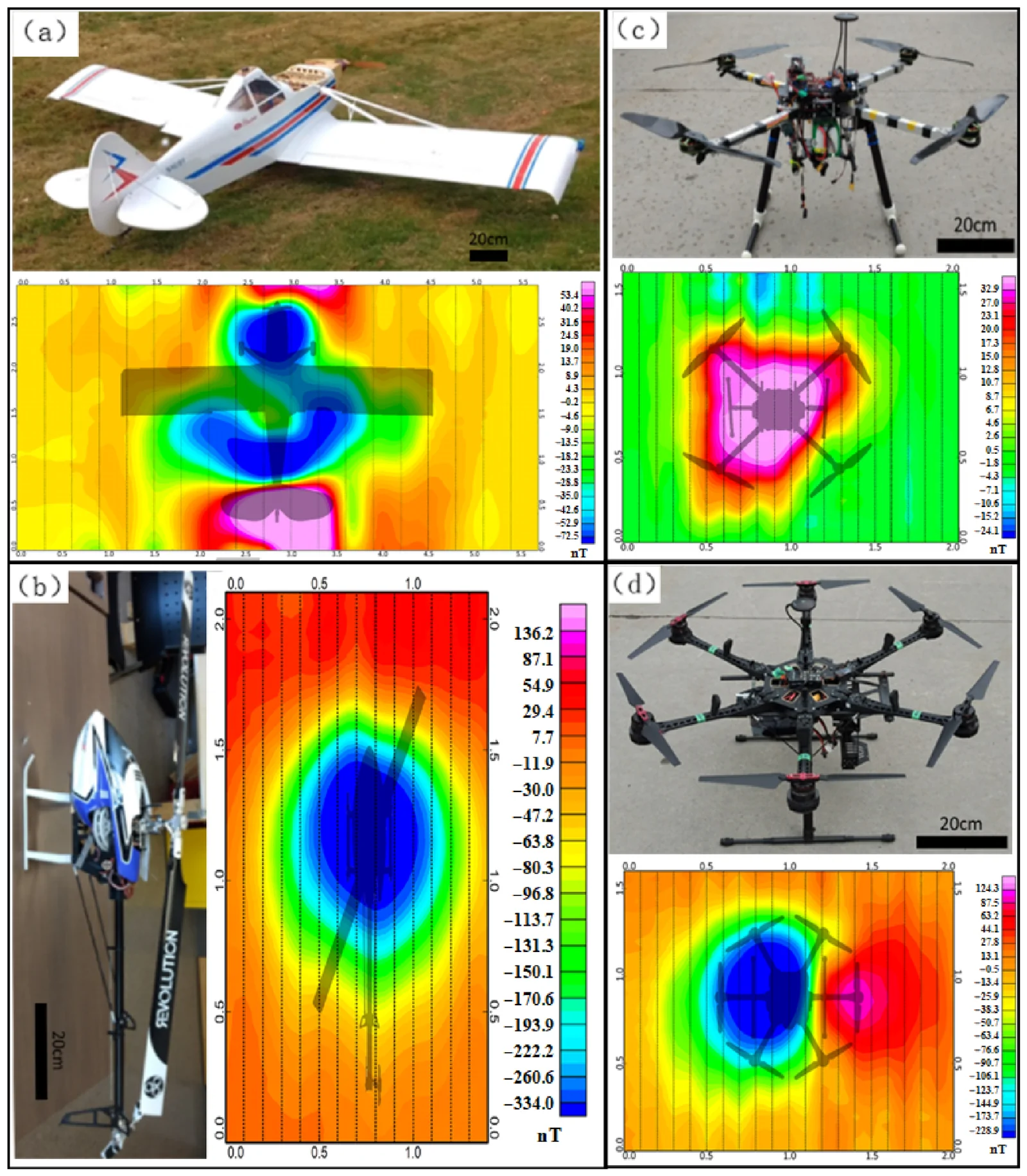
Four typical magnetic interference spectra of unmanned aerial vehicle systems for aeromagnetic measurements. (**a**) Single-motor fixed-wing UAV and its interference map; (**b**) Single-rotor helicopter and its interference map; (**c**) Quad-rotor helicopter and its interference map; (**d**) Hexa-rotor helicopter and its interference map.

With the vigorous growth of the aviation industry and the low-altitude economy, aviation geomagnetic navigation has become a research hotspot in the field of autonomous positioning for high-dynamic platforms, owing to its unique advantages of strong autonomy, all-weather operability, and immunity to interference. However, during high-speed flight, the relative attitude between the airframe and the geomagnetic field changes continuously and rapidly, while the residual, induced, and eddy-current magnetic fields generated by the platform itself—such as those from engines, electrical control systems, and magnetized structures—constitute a complex dynamic interference background. Experimental results indicate that the average magnetic interference intensity of typical aviation platforms, including single-motor fixed-wing aircraft and single-/multi-rotor unmanned aerial vehicles (UAVs), ranges from 3.1 to 7.4 nT, with interference peaks reaching as high as 21.4–574.2 nT [33] (as shown in Figure 4). Consequently, real-time accurate geomagnetic measurement and reference-map matching and filtering under strong interference have become the core technical bottleneck of aviation geomagnetic navigation. To overcome this challenge, numerous researchers have conducted long-term investigations from multiple perspectives, including hardware compensation, algorithmic optimization, and multi-source fusion.

**Figure 5:**
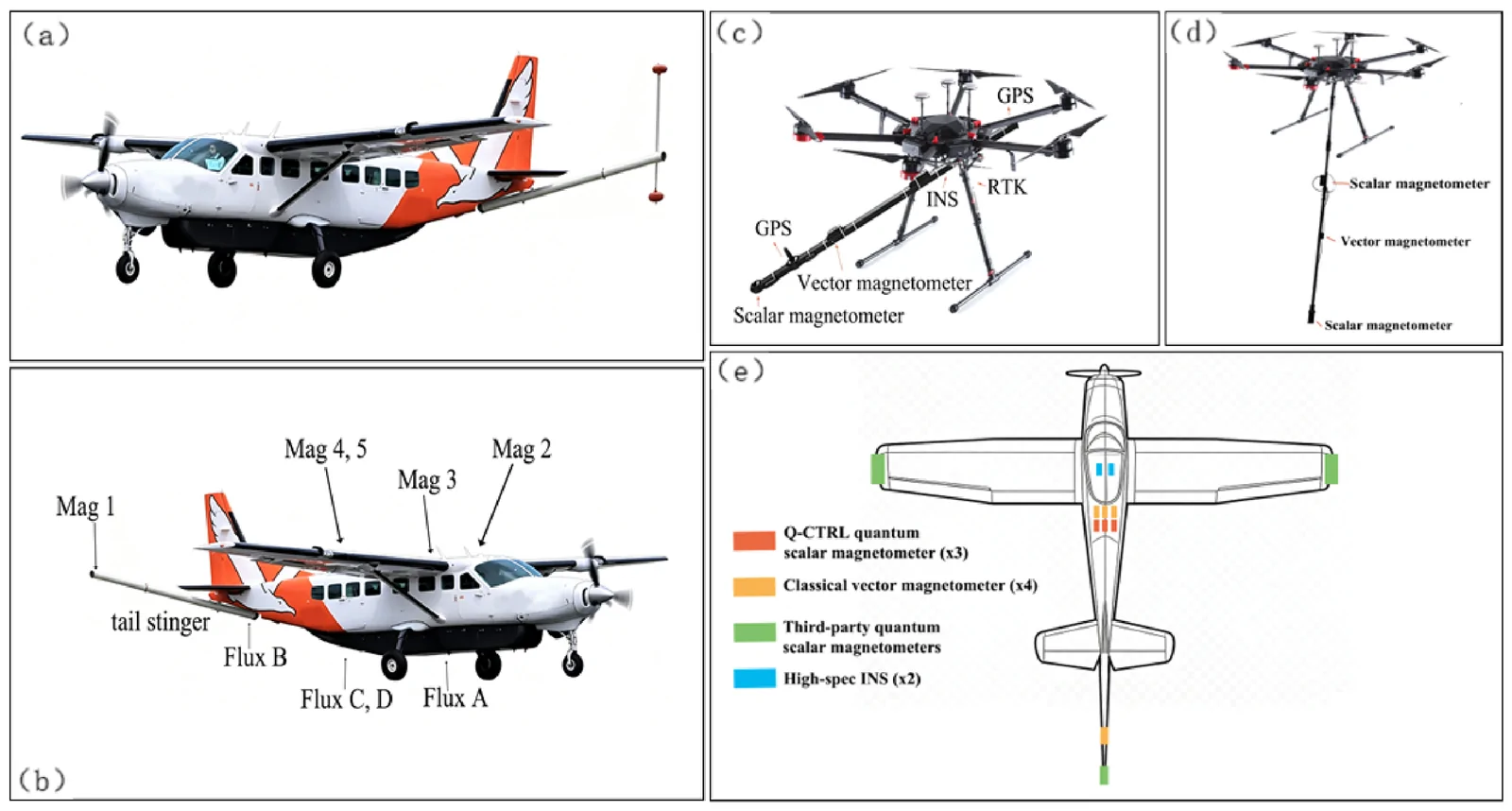
Geomagnetic navigation system for aircraft platforms. (**a**) Geomagnetic navigation system for manned aircraft, equipped with cesium light-pumping magnetometer, barometer, and inertial navigation system, etc.; (**b**) Geomagnetic navigation system for manned aircraft, equipped with multiple scalar and vector magnetometers, barometer, current-voltage sensors, and inertial navigation system, etc.; (**c**) Fixed-rod flight verification platform for rotorcraft (parallel to the ground), equipped with inertial navigation system, atomic scalar magnetometer, fluxgate magnetometer, etc.; (**d**) Fixed-rod flight verification platform for rotorcraft (can be lowered vertically below the ground), equipped with 2 light-pumping magnetometers and 1 fluxgate magnetometer; (**e**) Q-CTRL geomagnetic navigation system, equipped with multiple scalar and vector magnetometers, high-performance inertial navigation system, pitot tube, etc.

Historically, as early as the late 1940s, the Goodyear Corporation developed the Automatic Terrain Recognition and Navigation (ATRAN) system, which was equipped with a tri-axial magnetometer onboard the aircraft and achieved navigation by optimally matching the measured geomagnetic data against a geomagnetic reference-map database during flight. The system was validated in 1958 at Holloman Air Force Base in the United States [33,100]. E-Systems, a pioneer of geomagnetic navigation technology, first demonstrated the MAGCOM system, which navigates using geomagnetic anomaly data, in the mid-1960s, and subsequently introduced the Sandia Inertial Terrain-Aided Navigation (SITAN) system in 1978, claiming a navigation accuracy comparable to that of the GPS of that era [101,102]. Over the past decade, aviation geomagnetic navigation has advanced considerably through the deep integration of artificial intelligence, quantum sensing, and advanced filtering theory. Canciani and Raquet (2017) [103] from the Air Force Institute of Technology equipped a geophysical survey aircraft with an optically pumped cesium scalar magnetometer, navigation-grade inertial navigation system and barometer. Using a measured magnetic-anomaly map with an amplitude standard deviation of 104 nT, they performed an approximately one-hour flight test at 1000 ft mean sea level. With a marginalized particle filter, a horizontal distance root-mean-square error of 13 m was obtained. To address severe magnetometer-signal contamination on high-interference platforms, Gnadt (2022, 2023) [18,92] from the Massachusetts Institute of Technology systematically designed and compared multiple linear and nonlinear aeromagnetic compensation schemes. Real-world experiments were carried out with five scalar and four vector magnetometers installed on the SGL aircraft. The best geomagnetic navigation accuracy of approximately 25 m was achieved, and a magnetic-navigation challenge was launched on GitHub [104], whose codebase remains under active development (https://github.com/MIT-AI-Accelerator/MagNav.jl, accessed on 12 June 2026). Yang et al. (2023) [105] combined geomagnetic navigation algorithms with SLAM algorithms and designed and constructed a UAV flight verification platform, effectively validating the feasibility and robustness of the proposed algorithms. Liu et al. (2025) [23] conducted geomagnetic navigation experiments using UAVs equipped with scalar and vector magnetometers, improving the model compensation accuracy in cabin scenarios. Murat Muradoğlu et al. (2025) [21] from Q-CTRL conducted flight experiments aboard a manned aircraft equipped with the Q-CTRL geomagnetic navigation system, which incorporates quantum magnetometers, vector magnetometers, and a strategic-grade INS. The flight test was performed at an altitude of approximately 19,000 ft. For the first time, magnetic-anomaly-based navigation positioning was realized using publicly available magnetic anomaly maps (Total Magnetic Intensity (TMI) Grid of Australia 2019—7th Edition). The total flight distance accumulated exceeded 6700 km, with an optimal positioning accuracy of 22 m. Representative airborne geomagnetic navigation systems are illustrated in Figure 5. The sensor configuration of manned aircraft platforms ((a), (b)) refers to the experimental setups reported in Refs. [18,92,103]. The rotor-based verification platforms ((c), (d)) adopt the sensor schemes described in Refs. [23,105]. The quantum-enabled geomagnetic navigation system in (e) is based on the Q-CTRL solution presented in Ref. [21].

### 4.2. Marine and Underwater Platform Applications

The ocean covers approximately 71% of the Earth’s surface, and seawater strongly attenuates GNSS electromagnetic signals, rendering navigation means such as GNSS, visual, and celestial navigation nearly ineffective, while active sonar exposes the carrier’s position by radiating acoustic waves outward. In this context, geomagnetic navigation—characterized by its passivity, strong concealment, and errors that do not accumulate over time—has become an important supplementary means for long-endurance autonomous navigation of underwater vehicles, including remotely operated vehicles (ROVs), autonomous underwater vehicles (AUVs), and submarines [12,13].

Marine and underwater geomagnetic navigation is primarily realized through a geomagnetic/inertial navigation system (INS) integrated architecture. This architecture exploits the long-term stability of geomagnetic information as an observation reference to constrain and correct the divergent accumulated errors of the INS; conversely, the INS leverages its short-term high-accuracy dead-reckoning capability to maintain the initial position estimate required for geomagnetic map search and to provide continuous navigation information between successive geomagnetic corrections [24,106]. However, the underwater environment poses severe challenges to geomagnetic measurement. First, the geomagnetic field intensity attenuates significantly underwater: according to empirical formulas, the region within 20 m of a magnetic source is a rapid-variation zone, where the field can decay to as little as one ten-thousandth of the source strength; as the diving depth increases, the anomaly-field signal weakens further, sharply degrading the signal-to-noise ratio (SNR) of the measurements [107]. Second, when surveys are conducted with a towed magnetometer, the sensor (tow fish) is towed behind the vessel via a cable, generally at a distance of at least two to three times the ship length [27], to avoid the magnetic interference of the hull; however, currents, waves, and wind can cause attitude instability and swinging of the towed body, producing unmodeled measurement errors. Furthermore, the scarcity of high-accuracy underwater geomagnetic reference maps remains a critical bottleneck: many sea areas are still survey blind spots, and existing publicly available global models cannot satisfy navigation requirements.

**Figure 6:**
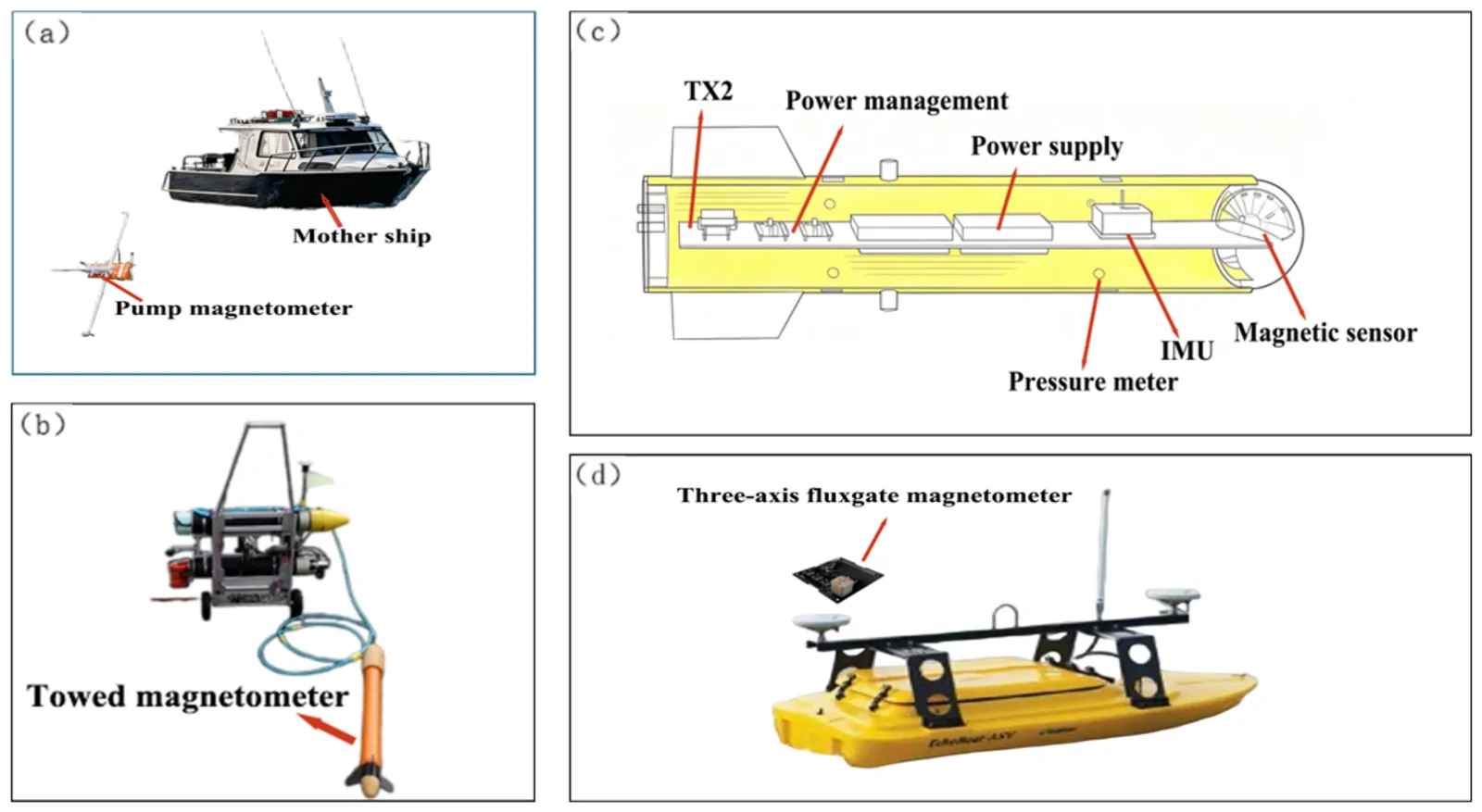
Marine and underwater magnetic navigation systems. (**a**) Magnetic-assisted navigation system based on ROV, equipped with a mother ship, including cesium light pump magnetometer, short baseline underwater positioning system, bathymetric sonar, depth gauge and magnetic compass, etc.; (**b**) Magnetic navigation system based on AUV, equipped with a towed magnetometer (towing distance 5 m to reduce magnetic interference); (**c**) Magnetic navigation system based on AUV, with built-in magnetometer, inertial measurement unit, pressure sensor, etc.; (**d**) Unmanned vessel cluster magnetic matching cooperative positioning system, equipped with three-axis fluxgate magnetometer.

To this end, numerous validation experiments on marine and underwater geomagnetic navigation have been conducted worldwide. In the 1980s, Plovani [108] of the United States attempted to employ magnetic navigation technology to achieve high-accuracy return to specific seabed locations; Carl Tyrén of Lund University, Sweden, conducted experimental validation of geomagnetic navigation technology using surface vessels [109]. In the early 21st century, the United States and Russia applied geomagnetic navigation to missile guidance and underwater vehicles, with the US systems achieving navigation accuracies better than 30 m in aerial environments and 500 m in underwater environments [110]. Zhao et al. (2008) [111] of Wuhan University completed an underwater geomagnetic navigation experiment in a sea area of Bohai Bay based on an improved TERCOM algorithm, achieving a local positioning accuracy of 100 m. Lin (2010) [112] of Peking University established a theoretical model and matching algorithms for underwater geomagnetic navigation and validated the feasibility of the method through experiments with field-measured data, attaining a positioning accuracy of approximately 400 m. In recent years, the National University of Defense Technology [64,113], Southeast University [114], and Wuhan University [106,115] have all conducted research on underwater geomagnetic navigation, though predominantly through simulation experiments. Nevertheless, a number of researchers have also carried out at-sea experiments for validation. In 2016, Zhang (2017) [116] of Harbin Engineering University conducted an ROV-based underwater geomagnetic-aided navigation experiment in the waters of Songhua Lake, effectively correcting the accumulated errors of the inertial navigation system. Martins da Cruz (2019) [117] constructed a loosely coupled geomagnetic navigation system based on a Medusa-class AUV, integrating a towed Overhauser magnetometer with a tow distance of 5 m, and conducted sea trials in Portuguese waters; by comparing the EKF, UKF, and PF nonlinear filters through 100 Monte Carlo simulations, the results showed that the PF achieved a divergence rate of only 1% under an initial covariance of 25 m^2^ (compared with 46% for the EKF and 39% for the UKF), and a geomagnetic navigation toolbox was developed. Yu et al. (2023) [118] proposed a geomagnetic positioning method based on the adaptive split-particle filter. Using a magnetic sensor array mounted on an AUV, combined with a self-surveyed geomagnetic map with a 50 m resolution of the experimental sea area, they conducted sea trials in Jiaozhou Bay, Qingdao, achieving an average positioning accuracy of approximately 546.44 m. Tang et al. (2024) [119] proposed a factor-graph-based geomagnetic matching cooperative positioning method for unmanned surface vehicle (USV) clusters (GUCP); the USVs were equipped with tri-axial fluxgate magnetometers, and sea trials were conducted in the Xisha Islands. The experimental results demonstrated that the method extends the operational duration of USV clusters while effectively improving positioning accuracy and stability. Gidugu et al. (2024) [120] from the National Institute of Ocean Technology, India, utilized 50 m-resolution prior geomagnetic data for a 900 km^2^ sea area generated from the NOAA World Magnetic Model. They adopted random-forest regression and decision-tree algorithms for trajectory guidance of an AUV equipped with a magnetometer of 0.1 nT sensitivity, and achieved a positioning accuracy of approximately 50 m in the two-dimensional plane. Li (2025) [121] of Harbin Engineering University, inspired by the geomagnetic and inertial navigation mechanisms found in biological navigation, developed a small AUV prototype employing micro-inertial/geomagnetic bionic navigation and conducted ocean experiments; an adaptive split particle filter-based geomagnetic positioning method was proposed, effectively realizing underwater navigation correction. Figure 6 illustrates marine and underwater geomagnetic navigation systems, in which the sensors include both towed and platform-integrated magnetometers, and the platforms include unmanned surface vessels, ROVs, and AUVs. The system design and sensor-deployment schemes for these marine platforms are documented in Refs. [110,111,112,113].

### 4.3. Indoor Applications

With the rapid advancement of urbanization and the increasing complexity of large indoor spaces—such as shopping malls, airports, subway stations, and underground parking garages—the demand for indoor positioning and navigation services has grown explosively. However, because buildings shield and attenuate satellite signals, GNSS cannot provide effective and reliable positioning services in indoor environments, and this technical bottleneck has spurred the research and application of a variety of indoor positioning technologies. Among these, geomagnetic positioning stands out for its unique advantages of requiring no additional infrastructure, exhibiting no accumulated error, and remaining unaffected by occlusion from ordinary objects, and it has gradually become one of the research hotspots. Indoor geomagnetic positioning exploits the magnetically anomalous fields with pronounced spatial specificity that are generated by the ferromagnetic materials (e.g., steel beams and rebar), electrical wiring, and various metallic facilities widely present in modern buildings. When superimposed on the Earth’s intrinsic magnetic field, these anomaly fields give rise to geomagnetic feature information that is strongly correlated with indoor position, thereby enabling positioning through feature-database matching [122,123,124].

**Figure 7:**
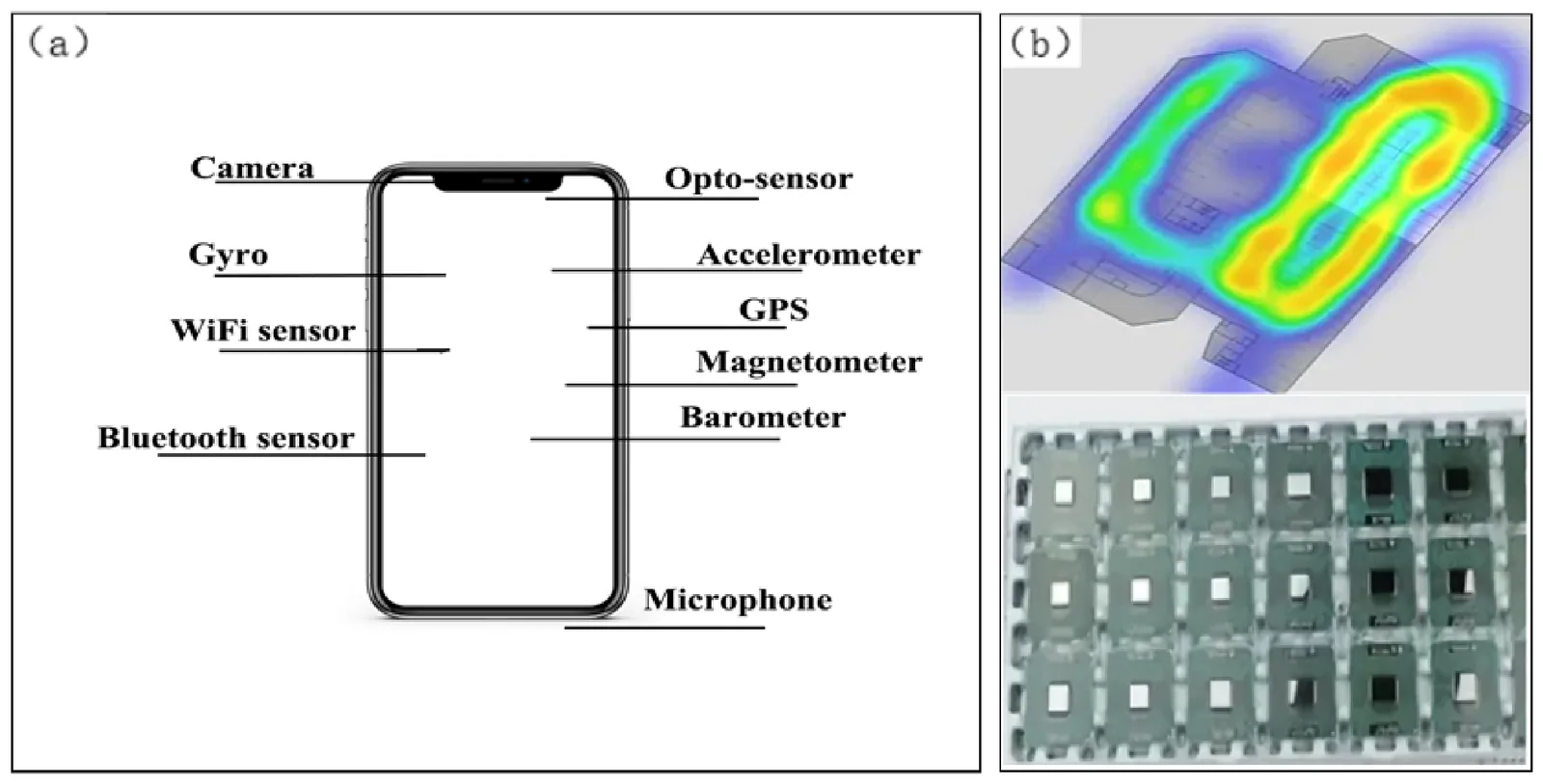
Indoor Magnetic Navigation System. (**a**) Smartphone sensors. It includes multiple sensors such as magnetometer, gyroscope, accelerometer, barometer, etc.; (**b**) Magnetic field maps and sensor array of the MAINS system. The upper part of the picture shows the schematic diagram of the magnetic field intensity variation inside the building, and the lower part shows the sensor board used in the experiment, which contains 30 PNI RM3100 magnetometers and one Osmium MIMU 4844 inertial measurement unit (IMU).

Unlike aviation and underwater applications, indoor positioning places greater emphasis on sensor solutions that are compact, low-power, and cost-controllable. Magnetoresistive sensors and Hall-effect sensors, owing to their ease of integration into consumer-grade products such as smartphones and wearable devices, have become the prevailing hardware choices for indoor magnetic sensing. This trend has also driven the transformation of geomagnetic positioning from professional surveying toward large-scale commercial applications. Representative indoor-oriented geomagnetic navigation-related platforms and test facilities are illustrated in Figure 7. Figure 7a demonstrates the multi-sensor suite integrated within consumer-grade smartphones, whose typical sensor composition is documented in Ref. [119].

At the academic research level, extensive studies have been devoted to multi-source information fusion combining geomagnetism with inertial navigation, Wi-Fi, and vision, and advanced algorithms such as deep learning, factor graph optimization, and adaptive filtering have significantly improved the accuracy and robustness of positioning systems. Lu et al. (2020) [125], based on the Android smartphone platform, fused geomagnetic, Wi-Fi, and pedestrian dead reckoning (PDR) technologies, applying an adaptive particle filter model and the random sample consensus (RANSAC) algorithm for weighted fusion of the collected signals; the indoor positioning error was minimized to 1.02 m, validating the significant advantage of multi-source heterogeneous information fusion in suppressing accumulated bias. Shu et al. (2023) [126] proposed an indoor geomagnetic positioning method based on direction-aware multi-scale recurrent neural networks (DM-RNNs), which incorporates pedestrian moving-direction information into both fingerprint construction and the entire positioning process to enhance spatial discriminability, employs multi-scale RNNs to extract both the long-term dependencies and the fine-grained local mutation features of magnetic sequences, and adopts an ensemble learning mechanism to strengthen model robustness against noise. Huang et al. (2024) [127] proposed the magnetic-field-aided inertial navigation system (MAINS) for indoor navigation, which combines a 30-channel MEMS magnetometer array with an observability-constrained extended Kalman filter and achieves a position error of less than 3 m in most cases during a 2 min navigation session; Figure 7b shows the geomagnetic maps and sensor array involved in the MAINS system. Sun et al. (2024) [128], addressing the problems of smartphone-based pedestrian dead reckoning, such as susceptibility to navigation-mode interference and poor model adaptability, constructed a pedestrian navigation mode recognition model based on combined motion-axis IMU sensor data, proposed an adaptive gait recognition algorithm that accounts for navigation modes, and designed a geomagnetic/PDR integrated positioning model together with a Wi-Fi FTM/PDR tightly coupled positioning model. The fusion scheme based on the extended Kalman filter (EKF) achieves a positioning error better than 0.69 m with an average execution time of less than 0.1 s, demonstrating the potential for long-term deployment on smartphones and wide-area indoor applications.

In parallel, the commercialization of indoor geomagnetic positioning has also made notable progress, with a number of technology companies emerging in this field. The indoor positioning platform developed by IndoorAtlas of Finland realizes indoor navigation by fusing smartphone IMU and magnetometer data through algorithms such as particle filters, provides multi-platform software development kits (SDKs) (https://www.indooratlas.com/, accessed on 25 June 2026), and achieves a positioning accuracy of 1–2 m [41]. Oriient of Israel deeply integrates geomagnetic navigation with inertial navigation systems and has constructed an indoor geomagnetic map database covering scenarios such as shopping malls and airports (https://www.oriient.me/company/, accessed on 25 June 2026). GiPStech adopts a magnetic-field fusion positioning architecture that combines BLE signals with deep learning algorithms to achieve sub-meter to 2 m positioning in industrial scenarios (https://www.gipstech.com/en/, accessed on 25 June 2026). The VENUE indoor positioning system developed by the TDK group of Canada leverages the geomagnetic sensors embedded in smartphones and dedicated terminals to readily identify the floor, location, and other information of indoor venues, achieving a positioning accuracy of 1–3 m (https://trustedpositioning.tdk.com/, accessed on 25 June 2026).

Taken together, these companies and research outcomes demonstrate that the co-evolution of multi-source information fusion and consumer-grade sensing hardware is steadily driving indoor geomagnetic positioning beyond the accuracy bottleneck of single-source geomagnetic matching, toward a comprehensive indoor positioning system characterized by high accuracy, low cost, and wide coverage.

## 5. Prospects for Geomagnetic Navigation Development

### 5.1. High-Accuracy Magnetic Survey Data Acquisition and High-Resolution Geomagnetic Reference Map Construction

The positioning accuracy and operational stability of geomagnetic navigation rely on pure measured magnetic field data as well as high-precision and high-timeliness geomagnetic reference maps. Nevertheless, severe coupling of multi-source carrier magnetic interference and insufficient resolution and coverage of existing reference maps constitute the two core bottlenecks restricting the practical application of current geomagnetic navigation technology. At the magnetic measurement level, interference sources such as the carrier’s inherent magnetization, dynamic induced magnetic fields, and eddy-current effects are highly coupled with the geomagnetic signal, severely degrading the quality of measured data; breakthroughs can therefore be pursued synergistically from both hardware architecture and software algorithms. On the hardware side, the application of non-magnetic or weakly magnetic materials can be promoted, and the installation architecture of magnetometers can be optimized to increase the distance from interference sources; alternatively, proactive compensation can be achieved by pre-constructing magnetic interference maps of the carrier under both static and dynamic conditions. On the software side, beyond conventional magnetic interference models such as the T–L model, suppression methods that conform to platform characteristics and adapt to dynamic interference need to be developed to achieve precise compensation for static remanent magnetization, dynamic induced fields, and nonlinear interference under complex maneuvers. With regard to geomagnetic reference maps, the spatial resolution of magnetic maps directly determines the theoretical upper limit of geomagnetic navigation accuracy. However, current satellite magnetic survey data are limited by observation altitude, and downward continuation to the ground or underwater suffers from instability and accuracy degradation, making it difficult to directly satisfy navigation requirements. Conversely, although conventional close-range surveys by surface vessels, manned aircraft, or underwater vehicles can acquire local high-accuracy data, they face practical challenges such as insufficient coverage of ocean areas, numerous survey blind spots, and prohibitive operational costs. To address this, the Defense Innovation Unit (DIU) of the US Department of Defense launched the Geomagnetic Airborne Unmanned Survey System (GAUSS) project in January 2026, planning to autonomously develop magnetic navigation technology on multiple aerial platform prototypes and to fund multiple aerial magnetic survey platforms to perform transoceanic mapping at an altitude of 30,000 ft and fine surface scanning at 2000 ft, thereby balancing coverage and spatial resolution. In the future, the magnetic field data acquisition capabilities of ground vehicles, smartphones, civil aircraft, and various vessels can also be fully aggregated to establish a multi-tier nested, accuracy-adapted global and regional geomagnetic field model system, thereby providing high-resolution, high-currency reference map support for geomagnetic navigation.

### 5.2. Deep Multi-Source Information Fusion and Intelligent Navigation Algorithm Innovation

Affected by inherent limitations of geomagnetic navigation, including scarce geomagnetic features in weak-gradient regions and insufficient positioning robustness under complex interference environments, the single geomagnetic matching mode is greatly restricted in practical scenarios and cannot meet the stable navigation requirements of high-dynamic and complex disturbance conditions. Against this background, geomagnetic navigation is evolving from single physical-field matching toward collaborative multi-source information fusion. Given that geomagnetic navigation tends to suffer from accuracy degradation or even failure in feature-poor regions or under strong interference, it is necessary to construct a navigation framework that couples multiple physical fields—including geomagnetic, inertial, acoustic, visual, and gravity—and to achieve information complementarity and mutual error suppression through tightly coupled or loosely coupled modes. At the algorithmic level, end-to-end navigation architectures based on deep learning can be further developed to extract deep spatiotemporal features directly from raw magnetic measurement sequences and multimodal sensor data and map them to carrier positions, thereby avoiding the reliance of conventional matching algorithms on linearization assumptions of magnetic maps. Meanwhile, traditional physical models can be introduced to guide and constrain neural networks, combining carrier kinematic constraints and geomagnetic field attenuation models with data-driven methods to enhance model generalization and interpretability, thereby overcoming the performance bottlenecks of conventional filtering and matching algorithms in strongly nonlinear, non-Gaussian noise environments.

### 5.3. Engineering Application and Industrialization of Geomagnetic Navigation

Current geomagnetic navigation technology faces prominent challenges, including significant deviations between simulation performance and practical engineering conditions, inadequate reliability in complex dynamic scenarios, and an imperfect industrial and standardization system, which greatly restrict its practical implementation and large-scale application. Therefore, promoting the transition of geomagnetic navigation from the laboratory to large-scale engineering applications is the core demand and urgent task of the field. Currently, commercial applications are mainly concentrated in the indoor geomagnetic positioning and navigation domain, where companies such as IndoorAtlas, Oriient, GiPStech, and TDK have all launched corresponding products (see Section 3.3 for details), whereas commercial implementation cases in China remain relatively limited, with the “Ciyun” positioning system developed by Beijing Maiding Aite Technology Co., Ltd. still in an early stage of promotion. In the aviation and underwater application domains, the United States and Australia have established significant leading advantages through sustained support from major military programs. In 2021, the US Air Force completed a geomagnetic navigation accuracy test on an F-16 fighter jet, achieving a positioning error of 59 m at a flight altitude of 300 m above ground level [129]. Between 2023 and 2024, the US Department of the Air Force, in collaboration with SandboxAQ, mounted a quantum magnetic navigation module on a C-17 transport aircraft, completing initial flight tests in large-scale military exercises such as “Golden Phoenix” and “Mobility Guardian,” and in 2024 achieved, for the first time, arrival at a target coordinate relying solely on quantum geomagnetic navigation with GPS fully disabled [74]. In 2025, a nationwide large-scale validation conducted by Airbus Acubed in collaboration with SandboxAQ further demonstrated the maturity of this technology: its AQNav quantum geomagnetic navigation system met the RNP 2.0 (en-route flight) standard or above on the vast majority of flight segments, and reached the RNP 0.3 (commercial airport approach) standard on some segments [41]. In the same period, Australia’s Q-CTRL received a contract worth 38 million Australian dollars from the US Defense Advanced Research Projects Agency (DARPA) under the “ Robust Quantum Sensors (RoQS)” program; its 2025 flight experiments achieved a final positioning error of 112 m at an altitude of 3600 ft (equivalent to 0.024% of the flight distance), and positioning errors ranging from 0.14% to 0.6% over a 365 km flight at 19,000 ft, with the best accuracy reaching 22 m (approximately 0.006% of the flight distance) [21]. In China, however, geomagnetic navigation research remains mainly concentrated in theoretical derivation and small-scale experimental validation at a limited number of universities and research institutions, lacking support from systematic major programs; there is still room for improvement in sensor engineering, platform integration, field test scale, and standardized validation processes. In the future, major special projects should further serve as the driving force to promote the self-reliant, controllable, and large-scale application of geomagnetic navigation technology.

## 6. Conclusions

Geomagnetic navigation exploits the Earth’s intrinsic magnetic field to achieve passive autonomous positioning. Owing to its strong concealment, resistance to interference, and round-the-clock, all-weather coverage, it holds irreplaceable strategic value in GNSS-denied or GNSS-degraded environments. This paper provides a systematic review of the geomagnetic navigation technology system along four dimensions: measurement and modeling, algorithmic architectures, multi-domain applications, and development prospects. The principal contributions of this paper are as follows: (1) a systematic review of the performance characteristics of mainstream magnetometers, including optically pumped, proton, and fluxgate types, together with the specifications of several high-resolution global and regional geomagnetic field models; (2) a synthesis of the core principles and key features of four algorithm families—geomagnetic matching, geomagnetic filtering, bio-inspired navigation, and artificial intelligence-assisted navigation—together with a delineation of the evolutionary trajectory from conventional correlation-based matching to data-driven intelligent decision-making; (3) an in-depth analysis of the technical bottlenecks, representative experiments, and development trends across three major application domains—aviation, marine and underwater systems, and indoor environments—providing a reference for multi-domain deployment; and (4) an identification of future directions from the perspectives of magnetic survey data quality enhancement, multi-source information fusion, and engineering and industrialization.

Overall, in recent years, geomagnetic navigation has been advancing rapidly from theoretical validation toward engineering practice. Nevertheless, notable gaps persist in sensor engineering, platform integration, large-scale field testing, and industrial deployment. Looking ahead, dedicated national programs should be leveraged as the primary driving force to accelerate the expansion of geomagnetic navigation from relatively mature scenarios, such as indoor positioning, toward strategic domains, including GNSS-denied aviation navigation and long-endurance autonomous underwater navigation, thereby establishing a self-reliant and controllable multi-domain navigation capability system that provides reliable positioning solutions in complex environments.

## Figures and Tables

**Table 1:** Performance comparison of magnetometers commonly used in geomagnetic navigation.

Performance Metric	Optically Pumped Magnetometer	Proton Magnetometer	Fluxgate Magnetometer	Magnetoresistive Magnetometer
Measured quantity	Scalar	Scalar	Vector	Vector
Resolution/nT	0.0001~0.01	0.001~0.1	0.01~1	1~300
Weight/g	<1000	~2500	<1000	<10
Representative instruments	CS-3, G-882, MG03, LCT-2A, GB-4A,, GB-10, GSMP-35	CZM, JOM-5SF, GSM-19, G-857	Mag-13 MFG-2S, Mag-03	HMC1021S, AA002-02E, BMM350
Suitable platforms	Vehicle-, airborne-, shipborne-, and underground platforms	Ground-based platforms	Vehicle-, airborne-, shipborne-, spaceborne-, and ground-based platforms	Ground robot-, smartphone-, and pedestrian-based platforms
References	[27,29,30,32,33,34,35]	[12,36,37,38]	[33,39,40]	[13,41,42,43]

**Table 3:** Comparison and analysis of common magnetic field filtering algorithms.

Filtering Algorithm	Core Principle	Nonlinear Approximation Strategy	Noise-Distribution Assumption	Advantages	Limitations	References
KF	Linear optimal recursive estimation	Exact analytical solution	Gaussian	Computationally efficient; provably optimal	Applicable only to linear systems	[67,68]
EKF	Locally linearized recursive estimation	First-order Taylor expansion	Gaussian	Simple, mature, and widely used	Prone to divergence under strong nonlinearity; sensitive to initial conditions	[69,73]
UKF	Sigma-point recursive estimation	Unscented transform	Gaussian	Simple, fast-converging, and suitable for real-time implementation	Sensitive to initial conditions; limited disturbance rejection capability	[71,72]
PF	Monte Carlo particle approximation	Sequential importance sampling	Arbitrary	Strong adaptability to nonlinear and non-Gaussian systems	Computationally intensive; poor real-time performance; particle degeneracy	[73,74]
AKF	Noise-statistics-adaptive correction	Adaptive noise modeling	Gaussian	Adapts to unknown time-varying systems; reduces model mismatch	Parameter tuning is complex; difficult to balance computational cost and estimation accuracy	[75,76]
FF	Distributed multi-source fusion	Multiple local filters operating independently	Local/structured	Fault-tolerant fusion; high accuracy and strong reliability	Complex information allocation	[77,78]

## Data Availability

No new data were created or analyzed in this study. Data sharing is not applicable to this article.
